# Functional characterization of the second feedback loop in the circadian clock of the Antarctic krill *Euphausia superba*

**DOI:** 10.1186/s12915-024-02099-2

**Published:** 2024-12-23

**Authors:** Chiara Stefanelli, Davide Colaianni, Gabriella M. Mazzotta, Gabriele Sales, Cristiano Bertolucci, Bettina Meyer, Alberto Biscontin, Cristiano De Pittà

**Affiliations:** 1 Department of Biology, University of Padova, Padova, 35121 Italy; 2 Department of Life Sciences and Biotechnology, University of Ferrara, Ferrara, 44121 Italy; 3 Institute for Chemistry and Biology of the Marine Environment, Carl Von Ossietzky University Oldenburg, Oldenburg, 26129 Germany; 4 Helmholtz Institute for Functional Marine Biodiversity (HIFMB), Carl Von Ossietzky University Oldenburg, Oldenburg, 26129 Germany; 5 Section Polar Biological Oceanography, Alfred Wegener Institute Helmholtz Centre for Polar and Marine Research, Bremerhaven, 27570 Germany; 6 Department of Agricultural, Food, Environmental and Animal Sciences, University of Udine, Udine, 33100 Italy

**Keywords:** Circadian clock, Antarctic krill, *Euphausia superba*, Second feedback loop, *vrille*, *pdp1*

## Abstract

**Background:**

The Antarctic krill *Euphausia superba* is a keystone species in the Southern Ocean ecosystem. This crustacean has an ancestral clock whose main components have been identified and characterized in the past few years. However, the second feedback loop, modulating *clock* gene expression through two transcription factors, VRI and PDP1, has yet to be described. The presence of this second regulatory mechanism is suggested by the identification of its negative component, *vrille*, at the transcriptional level.

**Results:**

Here, we describe the second feedback loop of krill by identifying the positive component, *pdp1*, and functionally characterizing both *pdp1* and *vrille*. Starting from the online transcriptome database KrillDB^2^, we identified and cloned three putative *pdp1* sequences which were subsequently analyzed for tissue expression and functional activity using luciferase assays, individually and in combination with two *vrille* isoforms. Among the *pdp1* isoforms*, Espdp1*_3 displayed higher expression levels in relevant circadian districts than the other two. Furthermore, *Es*PDP1_3 and *Es*VRI_2 exhibited the expected positive and negative regulation of the V/P-box in our in vitro system. Finally, *Espdp1*_3 and *Esvrille* also showed rhythmic expression in light–dark cycles, supporting their involvement in the regulation of the main circadian clock of the Antarctic krill.

**Conclusions:**

This study expands our knowledge about the molecular architecture of the Antarctic krill circadian clock by defining the components that take part in the modulation of *clock* expression, establishing a second feedback loop.

**Supplementary Information:**

The online version contains supplementary material available at 10.1186/s12915-024-02099-2.

## Background

The Antarctic krill *Euphausia superba* is a high-latitude pelagic crustacean that plays a central role in the Southern Ocean food web as prey for a wide range of predators and as grazer on both autotrophic and heterotrophic plankton [1]. Its conspicuous biomass, estimated to be around 379 million tonnes [2], makes it one of the most abundant species in the world and the largest fishery by tonnage in the Southern Ocean [3]. Even though there are limitations in krill harvesting, its commercial interest is increasing. Moreover, climate change impacts its survival and, consequently, the entire Southern Ocean ecosystem [4]. The Antarctic krill also plays an important role in biogeochemical cycles by transporting and transforming essential nutrients [5]. This impact is amplified by krill’s diel vertical migration (DVM), during which krill migrates from the surface at night to the deeper ocean layers in the daytime in a rhythmic pattern. Such movement in the water column is widespread among many marine organisms, and the migration amplitude is primarily species-specific [6]. This rhythmic behavior is regulated by an endogenous circadian clock [7, 8], a molecular mechanism evolved by most living organisms to optimize their physiological and behavioral response towards daily and seasonal changes in their habitat [9]. Although differences occurred during evolution in the structure and function of the main factors in different species [10], molecular clocks mainly consist of overlapping transcription-translation feedback loops (TTFL) composed of positive and negative regulators. Those transcription factors generate a self-sustained molecular oscillation of about 24 h that is synchronized with the environment and persists even without an external cue, usually with a variation in the period length [9]. In the Antarctic krill, the main entrainment cue (*Zeitgeber*) is the photoperiod, which influences the daily regulation of different krill’s physiological functions, such as oxygen consumption and metabolic activity [11, 12].

The main components of krill’s circadian clock have been characterized, revealing the structure of an ancestral clock that shares features with mammalian and insect clocks. Indeed, as in the monarch butterfly *Danaus plexippus* [13], it possesses both a *Drosophila*-like cryptochrome CRY1 degraded by light, and a light-insensitive vertebrate-like cryptochrome CRY2 that inhibits the dimer formed by CLOCK and CYCLE (CLK:CYC), core components of the circadian clock [14, 15]. Those two proteins act as transcriptional activators of different clock output genes and their own inhibitors, creating a TTFL that also resembles the one of *D*. *plexippus*, with some differences [15]. For example, in *E. superba*, the CLK:CYC inhibition is mediated not only by the complex formed by CRY2, PERIOD (PER), and TIMELESS (TIM) but also by the PER:TIM dimer as in *Drosophila melanogaster* [15]. In addition to the main feedback loop, a second TTFL has been identified in *D. melanogaster*, based on two basic leucine zipper (bZip) transcription factors, Vrille (VRI) [16] and PAR domain protein 1 ε (PDP1ε) [17, 18]. Four proteins of the same superfamily (human hepatic leukemia factor, HLF; chicken vitellogenin protein/rat thyrotrophic factor, VBP/TEF; rat albumin D-box-binding protein, DBP) form a sub-family that possess a proline- and acid-rich (PAR) domain that is present also in PDP1 proteins [19]. Different isoforms in flies have been identified for the two transcription factors, with various roles and tissue specificity. In particular, five *vrille* transcripts produce two protein variants, named short VRI and long VRI, both involved in the circadian clock, with the short one having a role also in development and being predominant in the adult [20]. While for *pdp1*, 13 transcript variants are deposited in FlyBase [21], encoding for 11 unique polypeptides. PDP1 isoforms are present in different tissues, and the expression of distinct isoforms is likely to control developmental pathways at transcriptional level [22]. Between the known isoforms, PDP1ε is the one involved in the circadian clock in *D. melanogaster* [18]. In this molecular mechanism, the expression of *vrille* and *pdp1ε* is mediated by the binding of CLK:CYC to the E-box located in the promoter of their genes. VRI and PDP1ε recognize and bind a V/P-box in the *clock* gene promoter and regulate its expression. Specifically, VRI inhibits *clock* expression, whereas PDP1ε acts as a transcriptional activator. The effect on *clock* expression appears to be delayed due to a combination of a stronger promoter and a shorter mRNA half-life for *vrille*. While *pdp1ε* mRNA is produced gradually, VRI reaches the level necessary to exert its influence on the *clock* promoter. Once PDP1ε reaches a similar level, VRI levels are already decreasing in response to the rapid degradation of its mRNA [18]. We have recently reported the rhythmic expression of the *E. superba* orthologue of *vrille* in light–dark (LD) and constant darkness (DD) conditions in both brain and eyestalk tissues [8, 23]. Moreover, Hunt et al. [24] reported the presence of a *pdp1* orthologue in the Antarctic krill transcriptome.

Starting from the most complete transcriptomic database on *E. superba*, KrillDB^2^ [25], we have identified and cloned three putative *pdp1* sequences. The analysis of the tissue-specific expression pattern, the functional activity, and the temporal expression in the head of *pdp1* and *vrille* candidates enabled the identification of the most likely components of the second circadian feedback loop in krill.

## Results

The endogenous clock of the Antarctic krill is one of the few ancestral clocks identified so far. We aimed to further characterize its regulatory components, starting from the hypothesis that clock components are organized in two interlocked feedback loops, reinforced by the identification of the negative regulator *vrille* [15]. This study focused on the identification of *pdp1* and the functional characterization of the second TTFL in *E. superba*.

### Sequence identification and description

Screening of the KrillDB^2^ with input sequences of known isoforms from *Drosophila pdp1* revealed different transcripts stemming from 5 distinct genes (gene ID: *ESG043930*, *ESG044741*, *ESG049747*, *ESG063279*, *ESG036078*; Additional file 1: Table S1 and Additional file 2: Table S2). Due to the conserved domains shared between *vrille* and *pdp1*, one of the results has previously been annotated as *vrille* (*ESG036078*) and was therefore eliminated as a potential *pdp1* sequence. Furthermore, gene *ESG063279* was excluded because there was no significant similarity with *Drosophila* sequences at the protein level.

In the end, three *pdp1* candidates in *E. superba* are reported in this study, referred as *Espdp1*_1 (ESG043930), *Espdp1*_2 (ESG044741), and *Espdp1*_3 (ESG049747).

**Fig. 1:**
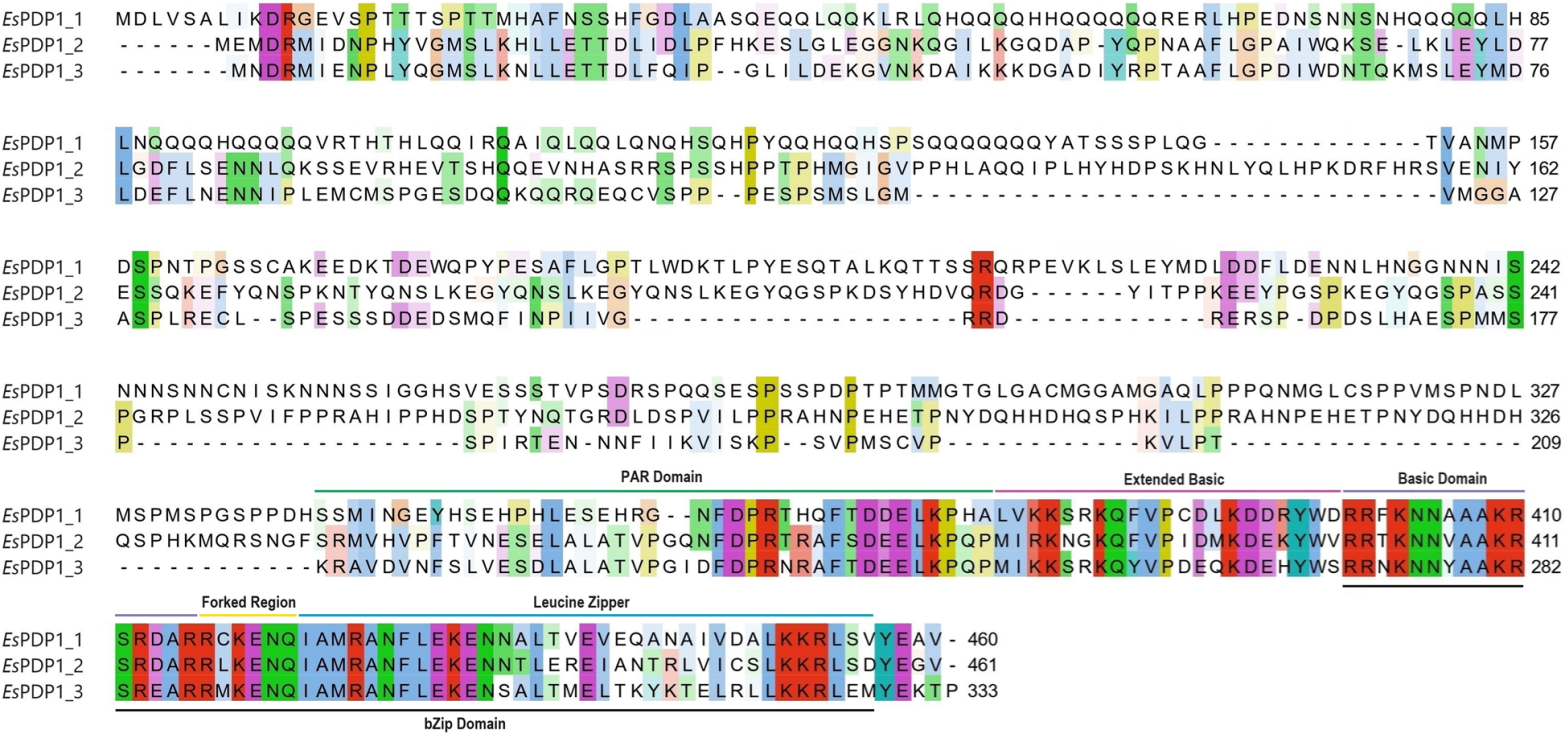
Multiple alignment of the three PDP1 candidates in *E. superba*. Functional domains are indicated above and below the multi-alignment based on Lin et al. [27]. The amino acid color was assigned by Jalview using the ClustalW color scheme, shaded by the degree of conservation

All these sequences shared high homology in the C-terminus region, where the functional domains are located, and exhibited greater variability in the N-terminus region, which is not associated with any specific function [22] (Fig. 1). The bZip domain was located at the C-terminus, comprising a basic domain, a forked region, and a leucine zipper domain, which are responsible for DNA binding and dimerization. Upstream the basic domain, an extended basic domain as well as a part of the PAR domain could be identified. The exact role of the last two domains remains to be definitively determined; however, they potentially participate in protein transactivation [26].

**Fig. 2:**
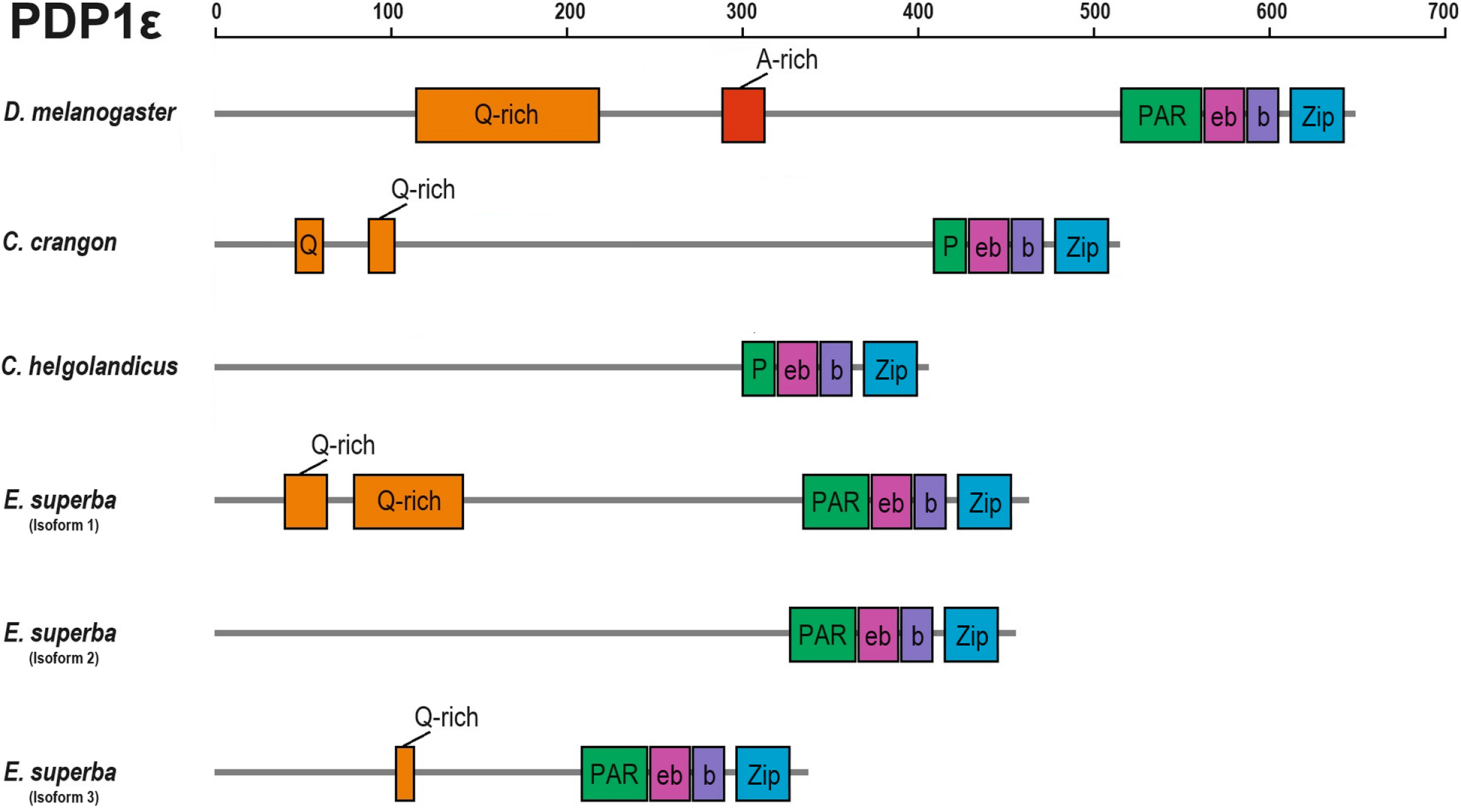
Schematic representation of the three putative PDP1 sequences in *E. superba* alongside known PDP1 isoforms. Functional domains and motifs are annotated based on Lin et al. [27]. bZip domain was confirmed by SMART analysis. Grey bars represent the protein length. Different colors and abbreviations are used to indicate each domain and motifs: glutamine rich (Q-rich) in orange, alanine-rich (A-rich) in red, PAR domain (PAR) in green, extended basic domain (eb) in dark pink, basic domain (b) in purple, and leucine zipper domain (Zip) in light blue. The krill sequences are compared to the PDP1 proteins of *D. melanogaster* (NCBI: NP_729302.2) [18], *C. crangon* (DN8383), and *C. helgolandicus* (DN4162) [28]

This peculiar domain organization was conserved across various PDP1 proteins from different species (Fig. 2). The recently discovered *pdp1* orthologs in zooplankton species (e.g., *Crangon crangon* and *Calanus helgolandicus* [28]) exhibit varying lengths, all of which are shorter compared to the *Drosophila* isoform involved in the circadian clock, *pdp1ε* [18]. Additionally, the PAR domain found in crustaceans is reduced in length compared to *Drosophila*. As expected, phylogenetic analysis of PDP1/TEF proteins from vertebrates, molluscs, and crustaceans revealed that *E. superba* PDP1 clustered within the Crustacea clade and emerged as the sister species to the Decapoda homologs included in the tree (Additional file 3: Fig. S1).

### Tissue expression patterns

**Fig. 3:**
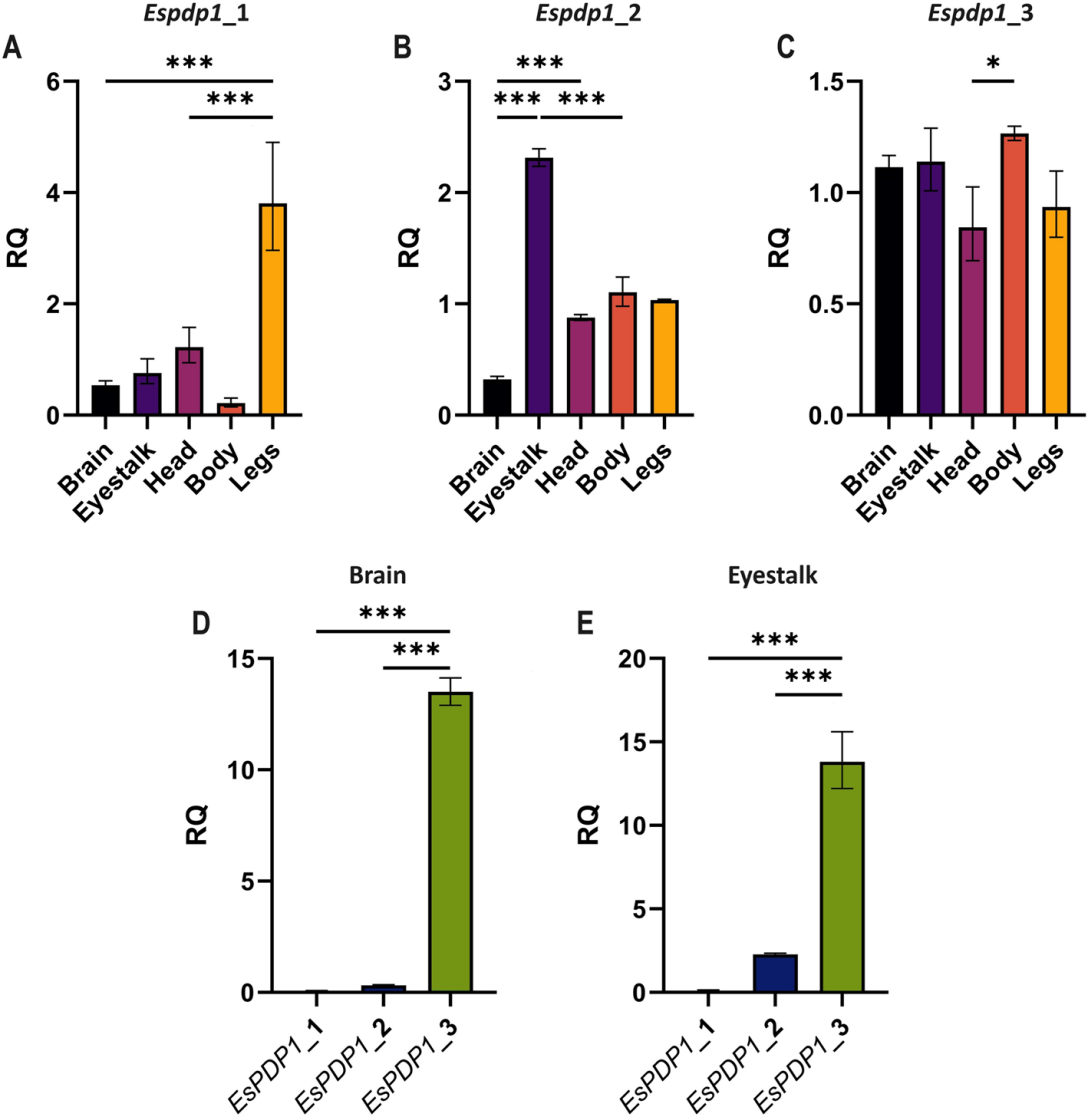
Relative expression levels (RQ) of (**A**) *Espdp1*_1, (**B**) *Espdp1*_2, and (**C**) *Espdp1*_3 across five different tissues of krill: brain, eyestalk, head, body, and legs. (**D**, **E)** Direct comparison of the expression levels of the three *pdp1* sequences in brain (**D**) and eyestalk (**E**) tissues. Data are represented as mean ± SD. *n* = 3 biological replicates. Statistical analysis was conducted using one-way ANOVA and Tukey’s post hoc analysis, with significance levels for the most meaningful comparisons denoted as *p < 0.05, **p < 0.01, and ***p < 0.005

The three *E. superba* sequences exhibited different expression patterns across different krill tissues. *Espdp1*_1 exhibited high expression levels in the legs while maintaining lower levels in other tested tissues (Fig. 3A). *Espdp1*_2 showed its highest expression in the eyestalk, with reduced levels in other tissues, particularly in the brain (Fig. 3B). Conversely, *Espdp1*_3, displayed ubiquitous expression across all analyzed tissues, with significant variation only between head and body districts (Fig. 3C). Comparative analysis of the three sequences within each tissue revealed that *Espdp1*_3 consistently exhibited the highest expression levels, even in the brain and eyestalk, where *Espdp1*_2 was primarily expressed, although not at levels significantly different from *Espdp1*_1 (Fig. 3D, E).

### Functional characterization

**Fig. 4:**
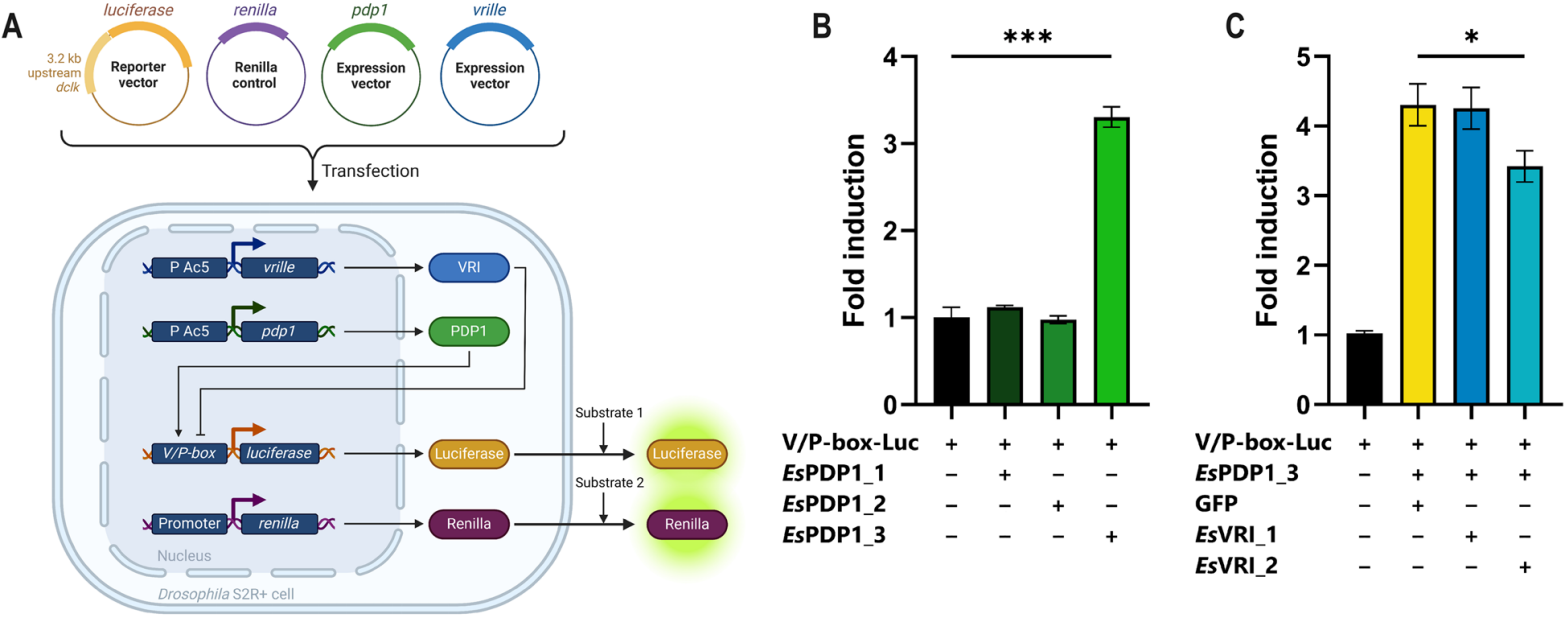
Functional characterization of the putative *Es*PDP1 and *Es*VRI activity. (**A)** Experimental setup in S2R+ transfected cells. The reporter vector brings the sequence encoding for luciferase with a 3.2-kb upstream sequence that is part of the *Drosophila clock* promoter [18]. The Renilla control vector is used for signal normalization. The activation of luciferase expression by PDP1 is inhibited by the site competition with VRI for the V/P-box (Created with BioRender.com). (**B)** Activation capability of the three PDP1 proteins individually expressed. (**C)** Inhibition activity of VRI proteins of the *dclk*/luciferase reporter induced by *Es*PDP1_3. The cells expressing both *Es*PDP1_3 and GFP proteins were used as positive control (see Methods). Negative control was set as 1. Data are represented as mean ± SEM. *n* = 3 independent transfections (biological replicates) were performed. Statistical analysis was performed using one-way ANOVA with Dunnett’s post hoc test, the statistical significance of the most meaningful comparisons is shown as *p < 0.05, **p < 0.01, and ***p < 0.005

To investigate the functional role of our candidates, we performed luciferase assays in *Drosophila* cells. Cells were transfected with expression vectors bringing the coding sequence of *vrille* and *pdp1* isoforms and luciferase reporter vector driven by a portion (3.2 kb) of the *Drosophila clock* (*dclk*) promoter containing 3 consensus sequences of the V/P-box [18]. A first experiment was designed to assess the activity of the three *pdp1* sequences. Subsequently, we conducted a second luciferase assay to evaluate the competition between PDP1 and VRI proteins for the V/P-box (Fig. 4A).

The *vrille* sequences encoded for proteins that differ by 15 amino acids at the N-terminus region. Specifically, the *Es*VRI_2 protein lacked the first 13 amino acids (MAAMMQSNVLQQQ) and two glutamines compared to the *Es*VRI_1 (Additional file 4: Fig. S2). Both proteins had a bZip domain, which was responsible for DNA binding.

In the first experiment, only *Es*PDP1_3 bound the V/P-box and induced luciferase expression. In contrast, the other two candidates exhibited no significant interaction with the reporter vector (Fig. 4B). Subsequent co-expression of *Es*PDP1_3 with each of the two VRI sequences revealed that only the shorter isoform *Es*VRI_2 inhibited luciferase expression induced by *Es*PDP1_3 through binding to the same site (Fig. 4C).

In conclusion, our experiments identified *Es*VRI_2 and *Es*PDP1_3 as putative components of the second feedback loop. Both bound to the V/P-box of the *Drosophila clock* promoter and regulated luciferase expression. The inhibitory action of *Es*VRI_2 was evident when it is co-expressed with the activator protein *Es*PDP1_3 and the competition for the same binding site resulted in a reduction of the luciferase signal. The observed mechanism aligns with the current knowledge in the in vivo modulation of *clock* expression by the two factors.

### Rhythmic expression

**Fig. 5:**
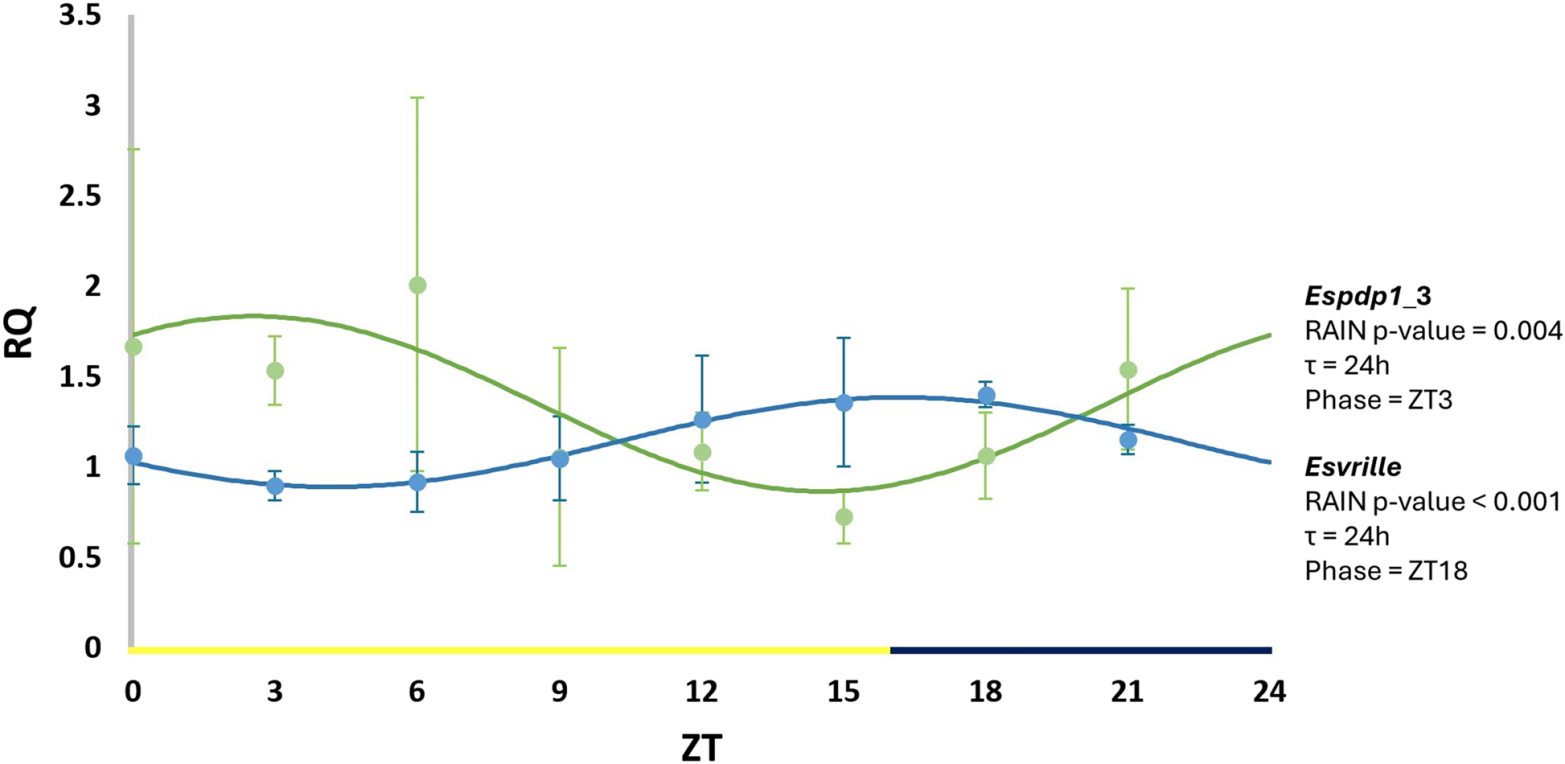
Temporal expression pattern for *Espdp1*_3 (green) and *Esvrille* (blue) in the head of krill sampled every 3 h in LD (16:8) conditions. Time is reported as *Zeitgeber* time (ZT). Relative quantification (RQ) is represented as mean ± SD. *n* = 4 (*Espdp1*_3) or 3 (*Esvrille*) distinct krill for each time point were used. Adjusted *p*-value, period (*τ*), and peak (phase) of the oscillation were estimated by RAIN algorithm. The sinusoidal curve that fit the data was represented using CircWave

We tested the temporal expression of *Espdp1*_3 and *Esvrille* in total RNA extracted from krill heads. A daily rhythmic pattern for both genes was confirmed by RAIN analysis [29] in krill sampled in LD conditions, with a clear temporal separation in their expression: *Espdp1*_3 reached a peak at the beginning of the day (ZT0-3), while *Esvrille* levels were maximum at the beginning of the dark phase (ZT18) (Fig. 5). The oscillation pattern did not show a pronounced amplitude, as in *Drosophila* [18]*.* This oscillation appeared to be influenced by light, as we did not observe a clear oscillatory pattern for *Espdp1*_3 under DD conditions (Additional file 5: Fig. S3). In contrast, *Esvrille* maintained a rhythmic expression even in DD conditions (Additional file 6: Fig. S4).

## Discussion

Living organisms possess an endogenous molecular clock that regulates their physiology and behavior, enabling them to adapt to upcoming environmental changes [9]. The molecular clock of *E. superba* represents an example of ancestral clock since it presents features of both *D. melanogaster* and mammal clocks [15]. The structure of the first feedback loop is known, and its regulation of biological rhythms has been observed in both metabolism and behavior [8, 11, 12]. In insects, a second loop, regulating *clock* mRNA level, has been observed [18]. This second feedback loop is composed of two distinct transcription factors, VRI and PDP1. The two proteins bind a V/P-box in the *clock* gene promoter and inhibit (VRI) or activate (PDP1) its transcription. The competition for the same binding site allows fine regulation to the mRNA oscillation of *clock*, a key component of the first loop [18]. In recent studies, the rhythmic expression of *Drosophila vrille* orthologue in *E. superba* have been observed [8]. Thus, a second feedback loop in the Antarctic krill is presumable.

The three *pdp1* cloned sequences possess a high similarity at the C-terminus region, in concomitance of the bZip domain, with a substantial difference at the N-terminus (Fig. 1). All the subdomains described for PAR domain proteins were inferred by literature [27], and part of the PAR domain was identified both in our sequences and in the newly annotated zooplankton species [28] (Fig. 2).

The phylogenetic analysis of the PAR proteins family also reveals that *E. superba* PDP1 is a homologous of the Crustacean TEF protein (Additional file 3: Fig. S1). Interestingly, previous investigations failed to identify any transcripts encoding PDP1 homologs in the copepods *Calanus finmarchicus* and *Tigriopus californicus* [30, 31]. Whether these members of the crustacean family possess the *pdp1* gene remains an intriguing and unresolved question.

The three candidate sequences for *pdp1* were differentially expressed in krill’s body districts (Fig. 3). Among these, *Espdp1*_3 likely plays a prominent role in the circadian clock. Its expression level is notably higher in the brain compared to the other two candidates (Fig. 3D). Additionally, it exhibits high expression in the eyestalk (Fig. 3E), where a peripheral clock of the krill is located [11]. While *Espdp1*_2 shows higher expression in the eyestalk compared to other regions (Fig. 3B), it does not reach the levels of *Espdp1*_3 and is comparable to *Espdp1*_1 (Fig. 3E). *Espdp1*_3 also displays high expression levels in other analyzed tissues (head, body, and legs) (Fig. 3C), with a significant reduction only in the head compared to the body. The lack of difference in expression levels between the head and brain or eyestalk tissues suggests that this reduction may result from the presence of more diverse tissues within the head, which could lower the overall expression level. The widespread expression of *Espdp1*_3 may indicate its involvement in mechanisms beyond the circadian system. In *D. melanogaster*, different isoforms of *pdp1* are expressed not only in the central nervous system but also in the developing embryo, in other tissues such as the epidermis, muscles, and fat body [22, 27], where the protein was initially shown to act as a regulator of Tropomyosin I gene expression in somatic body-wall and pharyngeal muscles [27]. Subsequently its role in the circadian clock was defined [18]. Accordingly, *Espdp1*_1 exhibits high expression levels in krill legs, suggesting a specific function at the muscular level (Fig. 3A).

Despite the functional domains at the C-terminus in all three candidates (partial PAR domain, extended basic domain, and bZip domain), only *Es*PDP1_3 could efficiently bind the *Drosophila* V/P-box (Fig. 4B). The three proteins differ in length and amino acid sequence, suggesting they likely have peculiar structures and different affinity for the binding site. Unfortunately, the *clock* promoter region in the recently published krill genome [32] is not well resolved, making it impossible to compare sites and test the specific V/P-box of the krill. However, given the high level of conservation of the V/P-boxes, we are confident in our findings regarding the activating action of *Es*PDP1_3 (Fig. 4B) and the inhibition of transcription mediated by *Es*VRI_2 (Fig. 4C) [15].

Furthermore, the expression patterns of *Espdp1*_3 and *Esvrille* show clear oscillation in LD conditions (Fig. 5), displaying a smoother trend compared to *Drosophila* orthologues [18]. This finding is consistent with previous observations for other clock genes in *E. superba* [8, 23]. For *Esvrille*, we observed a rhythmic expression also in DD conditions, in line with what was previously reported in brain and eyestalk tissues separately [8] (Additional file 6: Fig. S4). Unfortunately, we could not detect a distinct oscillation of *Espdp1*_3 under the same condition (Additional file 5: Fig. S3). However, this is a preliminary characterization of the temporal expression of the two circadian factors, focusing solely on their mRNA levels. Giving previous observations on the *D. melanogaster* homologs [18], quantifying their protein expression over a 24-h period would yield a more comprehensive picture.

## Conclusions

**Fig. 6:**
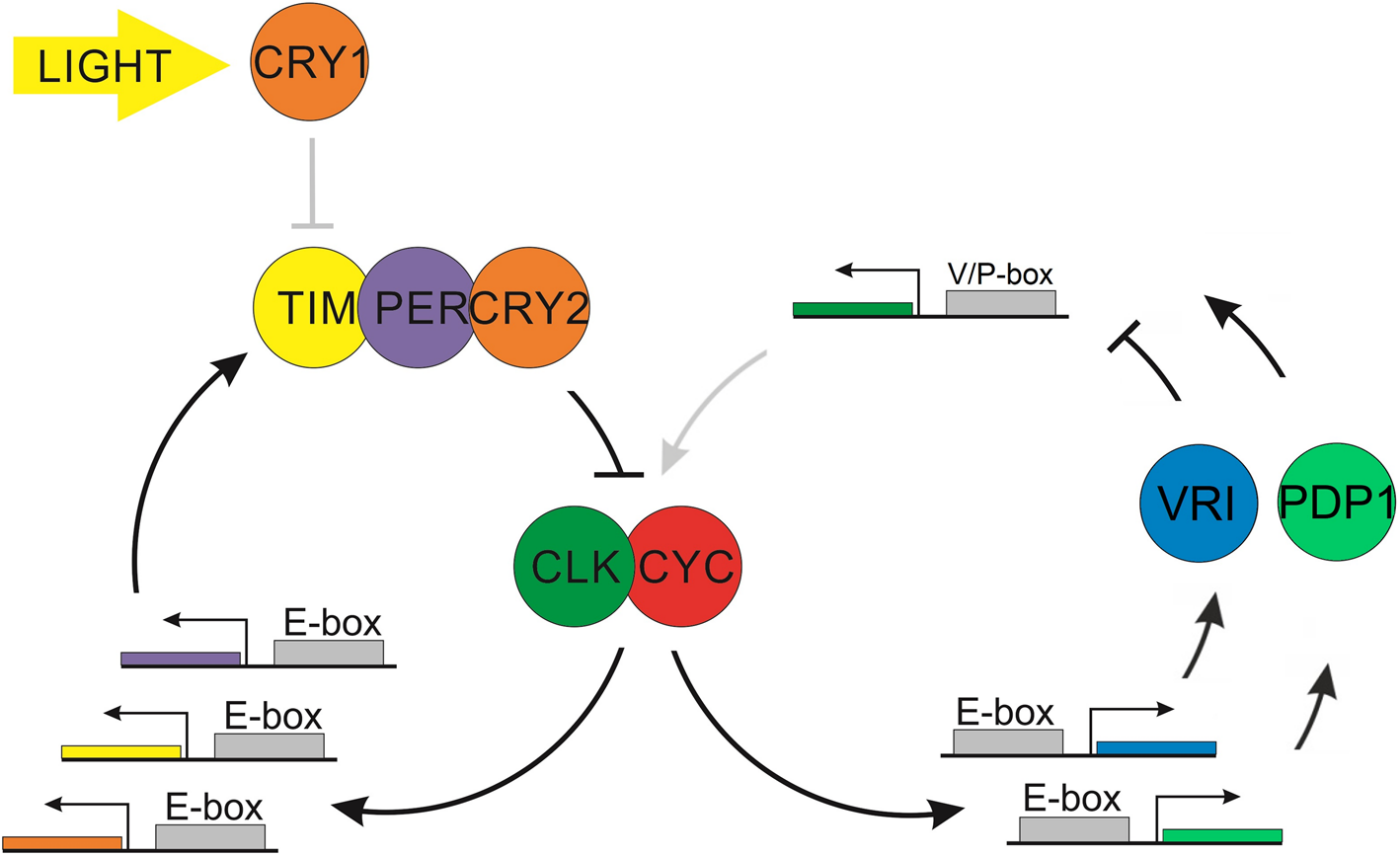
Schematic model of the two interlocked feedback loops of the circadian clock in *E. superba*. Verified interactions are represented with black arrows, while presumable interactions are in grey. The main loop (on the left) was characterized by Biscontin et al. [15], and it is composed of CLOCK (CLK), CYCLE (CYC), TIMELESS (TIM), PERIOD (PER), and CRYPTOCHROME 2 (CRY2) under the light entrainment mediated by CRYPTOCHROME 1 (CRY1) activity. The second loop (on the right) is composed of VRI and PDP1 transcription factors that inversely regulate *clock* transcription by binding the V/P-box located in its promoter region

This study expands our knowledge about the molecular architecture of the Antarctic krill circadian clock by validating a second feedback loop modulating *clock* expression. *Espdp1*_3 has a significant transactivation activity on the V/P-box that is attenuated by the competition with *Es*VRI_2, which has an inhibitory action. Moreover, *Espdp1*_3 is highly expressed in the brain and eyestalk, and shows a rhythmic expression pattern in krill heads in LD conditions. According to our results, we propose an updated model of the molecular structure of krill’s endogenous clock with two interlocked feedback loops, as in *Drosophila* (Fig. 6).

## Methods

### Sequence analysis and cloning

Candidate sequences coding for PDP1 were identified in the *Euphausia superba* transcriptome database KrillDB^2^ [25] using all the *Drosophila* known isoforms as inputs (Additional file 1: Table S1).

*E. superba pdp1* cDNA sequences were converted into amino acid sequences using the translate tool by ExPASy Proteomics (https://www.expasy.org/ [33]) and then aligned with other homologous sequences obtained from UniProtKB (http://www.uniprot.org/ [34]) using Clustal Omega v1.2.4 (http://www.ebi.ac.uk/Tools/msa/clustalo/ [35]). A phylogenetic tree was generated using a neighbor-joining algorithm based on the Jones-Taylor-Thornton (JTT) model (MEGA 11 [36]). Confidence in nodes was estimated by 1000 bootstrap replicates. A pairwise deletion algorithm was also used to eliminate any alignment gaps in the sequence. The tree was rooted using the *E. superba* pinopsin as outgroup.

Protein sequences were colored with Jalview v2.10.1 (http://www.jalview.org/ [37]) according to the default CLUSTALX conversion.

Primers for PCR amplification were designed with the web-tool Primer BLAST (http://www.ncbi.nlm.nih.gov/tools/primer-blast/) using all the possible variants for each of the putative sequences found as query (Additional file 2: Table S2 and Additional file 7: Table S3). cDNA was synthetized from a pool of previously extracted Total RNA [23] using GoScript Reverse Transcriptase (Promega, US) with oligo(dT) primer (Promega, US). Amplification of *pdp1* putative sequences was performed using GoTaq^®^ DNA Polymerase (Promega, US) and checked with gel electrophoresis. Following the manufacturer’s instruction, the amplified sequences were cloned using the StrataClone PCR Cloning Kit (Clontech, Japan) and then sequenced at BMR Genomics (Padova, Italy) with T7 and T3 primers. For each gene, we selected the cloned sequence with the highest percentage of identity compared to those already present in the database for further characterization.

### Constructs and S2R+ cells transcriptional activation assay

The putative *pdp1* coding sequences were subcloned into the S2 expression vector pAC5.1/V5-His A (Thermo Fischer Scientific, US) with the In-Fusion Snap Assembly cloning kit (Takara Bio, Japan). Primers for this step were designed with the In-Fusion Cloning Primer Design Tool (Additional file 7: Table S3). The vector was digested with *Eco*RI-HF^®^ and *Not*I-HF^®^ (NEB, US) restriction enzymes according to manufacturer’s instruction. Both PCR product and digested plasmid were purified with gel extraction with NucleoSpin^®^ Gel and PCR Clean-up (Macherey–Nagel, Germany). The same procedure was applied to two *vrille* isoforms, the one already published (GenBank Accession Number: KY923002.1), and a second one cloned in the same manner [15], reported in this work as isoform 1 and 2, respectively. The cloned sequences were additionally screened for polymerase synthesis errors through Sanger sequencing conducted at Eurofins (Essex, UK) using BGH reverse and AC5 primers.

*Drosophila* S2R+ cells (Invitrogen, US) were maintained at 25 °C in Schneider’s *Drosophila* medium (BioWest, France) with 10% serum. Approximately 4 × 10^5^ cells were transfected using Effectene^®^ Transfection Reagent (Qiagen, Germany) in 12-well plates. Constructs were transfected with a 3:1 molar ratio to 50 ng pCopia-Renilla (Addgene plasmid # 38093). The luciferase reporter vector was generously provided to us by Professor Justin Blau (Department of Biology, New York University, NY, US). It consists of a sequence encoding luciferase and an upstream 3.2-kb region that is part of the *Drosophila clock* promoter [18]. The total amount of transfected DNA was maintained constant across all the experimental conditions using the empty pAC5.1/V5-His A vector. Six independent transfections were performed for each condition. After 36 h, cells were harvested and processed following the protocol provided by the Dual Luciferase Reporter Assay Kit (Promega, US). Luciferase activity was measured with the DLReady Luminometer TD20/20 (Turner Designs, US) and normalized with Renilla signal. For each experiment, a negative control transfection (*dclk*-luc, pCopia-Renilla, pAC5.1/V5-His A) was used to establish the baseline reporter signal. In the case of multiple protein expressions, Ac5-STABLE2-neo (Addgene plasmid #32426) expressing GFP was added to the positive control to minimize variations in signal due to different commitments of the translational complex across the tested conditions. One-way ANOVA and Dunnett’s post hoc test were conducted using the GraphPad Prism Software (version 8.0.2) to determine the significance of comparisons. Overall, three biological replicates were performed.

### Quantitative real-time PCR

The expression levels of all the putative *pdp1* sequences were investigated across five distinct tissues of krill: head, brain, eyestalk, main body, and legs. cDNAs were obtained from a pool of previously extracted RNA [23] using GoScript^TM^ Reverse Transcriptase with random hexamers (Promega, US). Two endogenous controls have been used: *ubiquitin carboxyl-terminal hydrolase 46* (*Usp46*) [38] and *RNA polymerase I-specific transcription initiation factor RRN3 isoform 1* (*RRN3*) [23].

qRT-PCR was performed using ExcelTaq™ 2X Fast Q-PCR Master Mix (SMOBiO, US) with 10 ng of cDNA in 10 μL volume. Specific primer pairs for each putative *pdp1* sequence were designed using the web-tool Primer BLAST (Additional file 7: Table S3). The efficiency of primer pairs was assessed through standard curve analysis for each target, and the dissociation curves confirmed their specificity. All amplifications were performed in triplicate. The relative expression ratio (RQ) was calculated with the 2^−ΔΔCt^ method [39]. One-way ANOVA and Tukey’s post hoc analysis were used to compare the expression among tissues using the GraphPad Prism Software (version 8.0.2).

The rhythmic expression of *Espdp1*_3 was analyzed by qRT-PCR over two 24-h time series (LD and DD). Four samples of krill RNA were collected every 3 h at eight time points (ZT0, ZT3, ZT6, ZT9, ZT12, ZT15, ZT18, and ZT21) as described by Biscontin et al. [23]. For each time point, cDNA was synthesized from 1 μg of total RNA extracted from single krill heads under both LD (16:8) and DD conditions. The reaction and data analysis are described above. Rhythmicity in the LD and DD series was detected using the RAIN software package for R/Bioconductor with a Δt of 3 and the independent method [29]. RAIN results were further validated applying CircWave [40, 41] and MetaCycle [42] (Additional file 8: Table S4). Analogous time series were used to evaluate *vrille* rhythmic expression, with 3 krill specimens at each time point. In this case, it was not possible to discern the two available isoforms with the primer design. Sinusoidal trend lines were calculated according to best CircWave regression [40, 41].

## Supplementary Information

Additional file 1: Table S1. Putative PDP1 sequences found in KrillDB^2^ using *Drosophila* sequences as query.

Additional file 2: Table S2. Putative *pdp1* genes identified in KrillDB^2^ along with their associated transcripts.

Additional file 3: Fig. S1. Phylogenetic analysis of selected PDP1/TEF proteins shows that *Es*PDP1 clusterizes with other Crustacean orthologs. Scale bars indicate amino acid substitutions per site. The *E. superba* Pinopsin has been used as outgroup. Accession numbers: XP_037778882.1: *Penaeus monodon*, XP_064117072.1: *Macrobrachium nipponense*, XP_045612923.1: *Procambarus clarkii*, XP_053647350.1: *Cherax quadricarinatus*, XP_042211659.1: *Homarus americanus*, XP_033742026.1: *Pecten maximus*, XP_021364925.1: *Mizuhopecten yessoensis*, XP_063414312.1: *Mytilus trossulus*, XP_062612114.1: *Saccostrea cucullata*, XP_048778877.1: *Ostrea edulis*, XP_005104321.1: *Aplysia californica*, XP_052794455.1: *Mya arenaria*, Q92172: *Gallus gallus*, Q9JLC6: *Mus musculus*, Q9W722: *Danio rerio*, A0A142BLT2: *Euphausia superba* Peropsin. Parts of the figure were created with BioRender.com.

Additional file 4: Fig. S2. Schematic comparison of the amino acid sequences of the two VRI isoforms, highlighting the differences in the N-terminus region.

Additional file 5: Fig. S3. Temporal expression pattern for *Espdp1*_3 in krill heads sampled every 3 h under DD conditions, with time reported as *Zeitgeber* time (ZT). Four distinct krill were sampled for each time point (*n* = 4). The RAIN algorithm could not estimate the circadian parameters with statistical significance.

Additional file 6: Fig. S4. Temporal expression pattern for *Esvrille* in krill heads sampled every 3 h under DD conditions, with time reported as *Zeitgeber* time (ZT). Three distinct krill were sampled for each time point (*n* = 3). Adjusted *p*-value, period (τ), and peak (phase) of the oscillation were estimated by RAIN algorithm.

Additional file 7: Table S3. List of primers used for PCR amplification, In-Fusion cloning, and qRT-PCR.

Additional file 8: Table S4. Results of *Espdp1*_3 and *Esvrille* rhythmic expression analysis in LD and DD time series with MetaCycle (JTK, LS, and ARS) and CircWave algorithms.

## Data Availability

This published article and its supplementary information files include all data generated or analyzed during this study. Cloned sequences are deposited in GenBank: PQ041305 (*Espdp1*_2), PQ041306 (*Esvrille*_2), PQ041307 (*Espdp1*_3), PQ050700 (*Espdp1*_1).

## Abbreviations

bZip: Basic leucine zipper
CLK: Clock
CRY: Cryptochrome
CYC: Cycle
DBP: Rat albumin D-box binding protein
DD: Constant darkness
DVM: Diel vertical migration
HLF: Human hepatic leukemia factor
JJT: Jones-Taylor-Thornton
LD: Light-dark
PAR: Proline- and acid-rich
PDP1: PAR domain protein 1
PER: Period
RQ: Relative quantification
RRN3: RNA polymerase I-specific transcription initiation factor RRN3 isoform 1
TIM: Timeless
TTFL: Transcription-translation feedback loop
Usp46: Ubiquitin carboxyl-terminal hydrolase 46
VBP/TEF: Chicken vitellogenin protein/rat thyrotrophic factor
VRI: Vrille
ZT: *Zeitgeber* time
