# Supplementary material for: Functional characterization of the second feedback loop in the circadian clock of the Antarctic krill Euphausia superba
Source: BMC Biol. 2024 Dec 23;22:298. doi: 10.1186/s12915-024-02099-2 (PMC11668059; doi:10.1186/s12915-024-02099-2)
Supplement: Supplementary file 3 — Additional file 3: Fig. S1. Phylogenetic analysis of selected PDP1/TEF proteins shows that EsPDP1 clusterizes with other Crustacean orthologs. Scale bars indicate amino acid substitutions per site. The E. superba Pinopsin has been used as outgroup. Accession numbers: XP_037778882.1: Penaeus monodon, XP_064117072.1: Macrobrachium nipponense, XP_045612923.1: Procambarus clarkii, XP_053647350.1: Cherax quadricarinatus, XP_042211659.1: Homarus americanus, XP_033742026.1: Pecten maximus, XP_021364925.1: Mizuhopecten yessoensis, XP_063414312.1: Mytilus trossulus, XP_062612114.1: Saccostrea cucullata, XP_048778877.1: Ostrea edulis, XP_005104321.1: Aplysia californica, XP_052794455.1: Mya arenaria, Q92172: Gallus gallus, Q9JLC6: Mus musculus, Q9W722: Danio rerio, A0A142BLT2: Euphausia superba Peropsin. Parts of the figure were created with BioRender.com. [file 12915_2024_2099_MOESM3_ESM.docx]

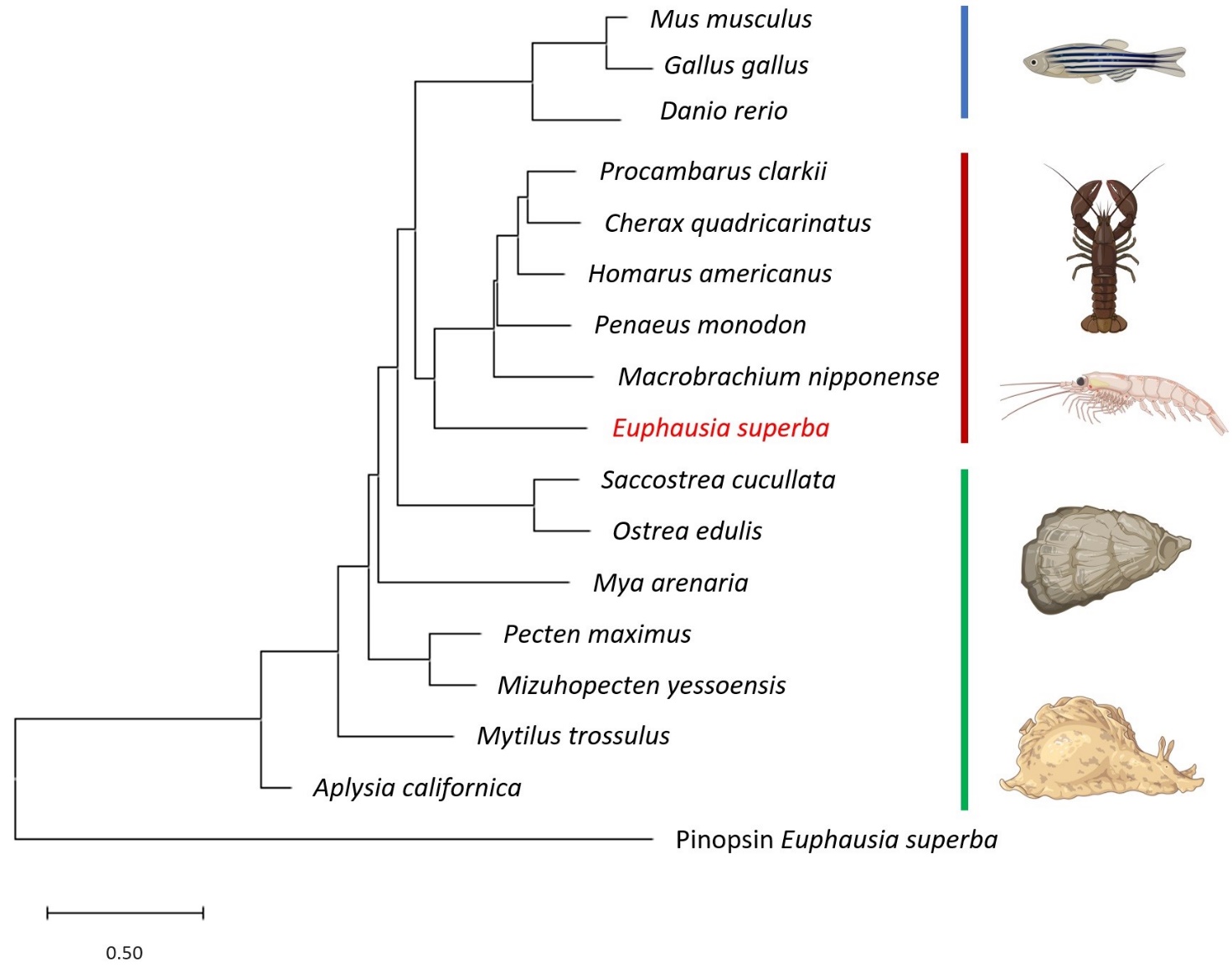


**Additional file 3: Fig. S1** Phylogenetic analysis of selected PDP1/TEF proteins shows that *Es*PDP1 clusterizes with other Crustacean orthologs. Scale bars indicate amino acid substitutions per site. The *E. superba* Pinopsin has been used as outgroup. Accession numbers: XP_037778882.1: *Penaeus monodon*, XP_064117072.1: *Macrobrachium nipponense*, XP_045612923.1: *Procambarus clarkii*, XP_053647350.1: *Cherax quadricarinatus*, XP_042211659.1: *Homarus americanus*, XP_033742026.1: *Pecten maximus*, XP_021364925.1: *Mizuhopecten yessoensis*, XP_063414312.1: *Mytilus trossulus*, XP_062612114.1: *Saccostrea cucullata*, XP_048778877.1: *Ostrea edulis*, XP_005104321.1: *Aplysia californica*, XP_052794455.1: *Mya arenaria*, Q92172: *Gallus gallus*, Q9JLC6: *Mus musculus*, Q9W722: *Danio rerio*, A0A142BLT2: *Euphausia superba* Peropsin. Parts of the figure were created with BioRender.com.
