# Supplementary material for: Functional characterization of the second feedback loop in the circadian clock of the Antarctic krill Euphausia superba
Source: BMC Biol. 2024 Dec 23;22:298. doi: 10.1186/s12915-024-02099-2 (PMC11668059; doi:10.1186/s12915-024-02099-2)
Supplement: Supplementary file 4 — Additional file 4: Fig. S2. Schematic comparison of the amino acid sequences of the two VRI isoforms, highlighting the differences in the N-terminus region. [file 12915_2024_2099_MOESM4_ESM.docx]

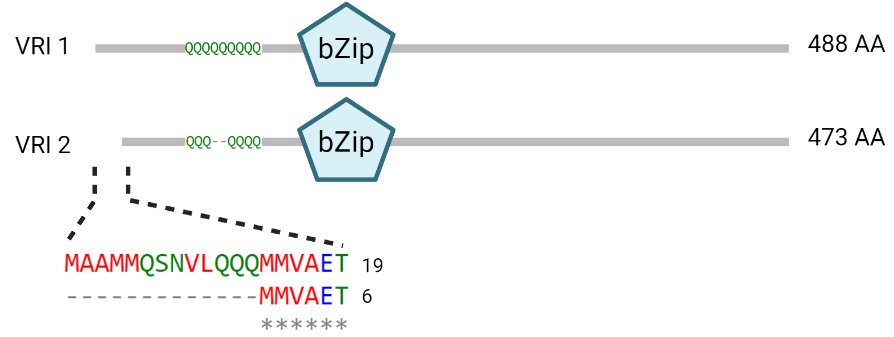


**Additional file 4: Fig. S2** Schematic comparison of the amino acid sequences of the two VRI isoforms, highlighting the differences in the N-terminus region.
