# Supplementary material for: Functional characterization of the second feedback loop in the circadian clock of the Antarctic krill Euphausia superba
Source: BMC Biol. 2024 Dec 23;22:298. doi: 10.1186/s12915-024-02099-2 (PMC11668059; doi:10.1186/s12915-024-02099-2)
Supplement: Supplementary file 5 — Additional file 5: Fig. S3. Temporal expression pattern for Espdp1_3 in krill heads sampled every 3 h under DD conditions, with time reported as Zeitgeber time (ZT). Four distinct krill were sampled for each time point (n = 4). The RAIN algorithm could not estimate the circadian parameters with statistical significance. [file 12915_2024_2099_MOESM5_ESM.docx]

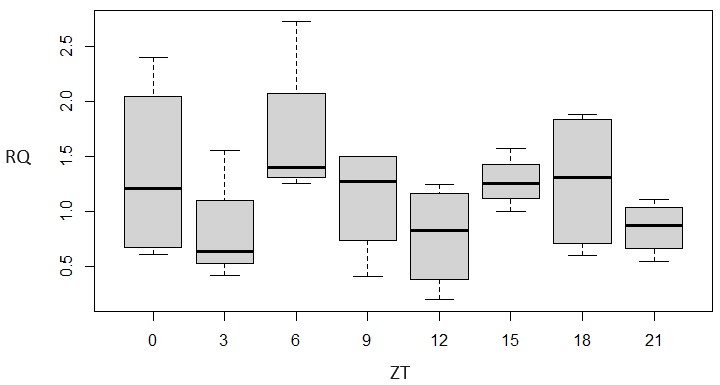


**Additional file 5: Fig. S3** Temporal expression pattern for *Espdp1*_3 in krill heads sampled every 3 hours under DD conditions, with time reported as *Zeitgeber* time (ZT). Four distinct krill were sampled for each time point (n=4). The RAIN algorithm could not estimate the circadian parameters with statistical significance.
