# Supplementary material for: Functional characterization of the second feedback loop in the circadian clock of the Antarctic krill Euphausia superba
Source: BMC Biol. 2024 Dec 23;22:298. doi: 10.1186/s12915-024-02099-2 (PMC11668059; doi:10.1186/s12915-024-02099-2)
Supplement: Supplementary file 6 — Additional file 6: Fig. S4. Temporal expression pattern for Esvrille in krill heads sampled every 3 h under DD conditions, with time reported as Zeitgeber time (ZT). Three distinct krill were sampled for each time point (n = 3). Adjusted p-value, period (τ), and peak (phase) of the oscillation were estimated by RAIN algorithm. [file 12915_2024_2099_MOESM6_ESM.docx]

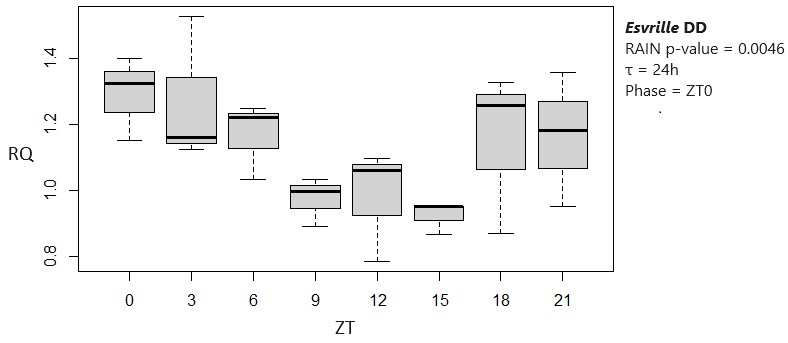


**Additional file 6: Fig. S4** Temporal expression pattern for *Esvrille* in krill heads sampled every 3 hours under DD conditions, with time reported as *Zeitgeber* time (ZT). Three distinct krill were sampled for each time point (n=3). Adjusted p-value, period (τ), and peak (phase) of the oscillation were estimated by RAIN algorithm.
